# Wound management, healing, and early prosthetic rehabilitation: Part 1 - A scoping review of healing and non-healing definitions

**DOI:** 10.33137/cpoj.v7i2.43715

**Published:** 2024-11-15

**Authors:** H Williams-Reid, A Johannesson, A Buis

**Affiliations:** 1 Department of Biomedical Engineering, Faculty of Engineering, University of Strathclyde, Glasgow, Scotland.; 2 Össur Clinics EMEA, Stockholm, Sweden.

**Keywords:** Amputation, Scoping Review, Wound Healing, Wound Non-Healing, Surgical Site Healing, Biomarkers, Markers of Healing, Residuum Healing, Residual Limb Healing, Wound Management, Impaired Healing.

## Abstract

**BACKGROUND:**

Following lower limb amputation, timely prosthetic fitting enhances mobility and quality of life. However, inconsistent definitions of surgical site healing complicate prosthesis readiness assessment and highlight the need for objective wound management measures.

**OBJECTIVE:**

This review aimed to compile definitions of healing and non-healing provided in the literature investigating biomarkers of healing of the tissues and structures found in the residual limbs of adults with amputation.

**METHODOLOGY:**

A scoping review was conducted following JBI and PRISMA-ScR guidance. Searches using “biomarkers,” “wound healing,” and “amputation” were performed on May 6, 2023, on Web of Science, Ovid MEDLINE, Ovid Embase, Scopus, Cochrane, PubMed, and CINAHL databases. Inclusion criteria were: 1) References to biomarkers and healing; 2) Residuum tissue healing; 3) Clear methodology with ethical approval; 4) Published from 2017 onwards. Articles were assessed for quality (QualSyst tool) and evidence level (JBI system).

**FINDINGS:**

Of 3,306 articles screened, 219 met the inclusion criteria and are reviewed in this article, with 77% rated strong quality. 43% of all included sources did not define healing, while the remainder used specific criteria including epithelialization (14%), wound size reduction (28%), gradings scales (3%), scarring (1%), absence of wound complications (2%), hydroxyproline levels (0.5%), no amputation (0.5%), or neovascularization (0.5%). 84% of included sources did not provide definitions of non-healing. Studies defining non-healing used criteria like wound complications (4%), the need for operative interventions (4%), or lack of wound size reduction (1%). For 10% of included sources, healing and non-healing definitions were considered not applicable given the research content. Total percentages exceed 100% for both healing and non-healing definitions because some sources used two definition classifications, such as epithelialization and wound size reduction. The findings indicate a lack of standardized definitions irrespective of study type.

**CONCLUSION:**

This review reveals significant gaps in current definitions of healing and non-healing, often based on superficial assessments that overlook deeper tissue healing and mechanical properties essential for prosthesis use. It emphasizes the need for comprehensive definitions incorporating biomarkers and psychosocial factors to improve wound management and post-amputation recovery.

## INTRODUCTION

### 1: OVERALL RATIONALE, AIMS, AND OBJECTIVES

The term “wound” broadly refers to damage to any biological tissue,^1^ encompassing damage from amputation surgery to deep tissue injuries caused by loading during lower limb prosthetic use. The healthy, or normal, wound healing process is marked by four interlinked physiologic phases (**Table 1**): I) hemostasis, II) inflammation, III) proliferation, and IV) tissue remodeling (or resolution).^2–4^ This complex process demands a high degree of cellular coordination, including several avenues through which impairments can occur. Consequently, wound healing can be stalled (also referred to as non-healing, impaired, or chronic) not by one isolated factor, but by several smaller contributing issues.^5^ For example, common post-amputation surgical site healing complications include infection, pain, hematomas, tissue necrosis, poor residual limb formation, recurrent ulceration, wound dehiscence, and stitch abscesses.^6,7^ Persistent complications, in other words, poor healing, can necessitate revision surgeries or even re-amputation at more proximal levels.^6^ Despite the intricacies of the wound healing process, the current assessment of healing relies mainly on surface level clinician examinations and wound classification systems. For instance, the East London NHS (National Health Service) Trust's clinical guidelines recommend using a disposable measuring tape to monitor wound healing by assessing wound length and width.^8^ Such subjective methods introduce biases and fail to account for underlying issues. Deep tissue injuries (DTIs), for example, develop subcutaneously and only become visible in later stages, manifesting as bruised purple localized areas of intact skin^9^ that can evolve into large deep wounds.^10^ This introduces the need for more objective measures to assess healing both at the surface level and below the cutaneous layer.

**Table 1*:** Characteristics and time frames of the four primary interlinked phases of wound healing.

Phase	Characterization	Time Frame
I (Hemostasis)	Directly after injury, there is an outpouring of lymphatic fluid and blood. This involves platelet aggregation (blood clotting) and blood vessel vasoconstriction to prevent further bleeding.	Seconds to Hours
II (Inflammation)	Cellular debris and bacteria are removed. Vascular permeability is increased to promote the diffusion of necessary molecules to the wound site. Cellular migration is similarly increased, as is chemotaxis. The aim is to limit further damage.	Hours to Days
III (Proliferation)	Formation of granulation tissue (the contractile organ that fills wounds that heal by second intention), reepithelization (epidermis regeneration), and neovascularization.	Days to Weeks
IV (Remodeling)	Defined by vascular maturation and regression, and collagen remodeling. The wound reaches its maximum strength and its ultimate endpoint; in cutaneous tissue, this is marked by a collagenous scar.	Weeks to Months

* Adapted from References 2–4.

This necessity for more objective measures is particularly pertinent in managing residual limbs following lower limb amputation. Following their surgery, depending on the healing process, individuals who have undergone lower limb amputation will typically receive a customized prosthetic limb within a window of 3 to 20 weeks post-surgery.^11,12^ These prosthetic interventions are bespoke devices aimed to replicate the missing limb function, enhancing the user's mobility, ambulation, and ability to perform daily tasks. Consequently, they significantly improve physical health, cardiovascular well-being, mental health, quality of life, and overall independence.^12,13^ Notably, Singh and Prasad^14^ reported that the absence of a prosthetic limb fitting is an independent predictor of mortality within three years of a major lower limb amputation, defined as the loss of the limb at or proximal to the ankle joint.^15^

However, assessing residuum healing and thus readiness for a prosthesis after amputation, like wound healing, remains ambiguous, involving clinician opinion, and surface level wound examination. In a narrative review of determinants of healing and readiness for prosthetic fitting after transtibial amputation, Day et al.^16^ concluded that clinical judgement is most subjective when assessing the degree of healing. Online resources for individuals with amputation similarly note that readiness for prosthetic fitting is dependent on factors such as healing, pain management, oedema, and residual limb volume,^17^ yet specific indicators for these factors remain undefined. Even healthcare bodies like the NHS provide no clear guidelines on assessing readiness, relying instead on clinicians' experience and judgement, which can vary widely. For instance, Turner et al.^18^ in their thematic analysis of issues faced by prosthetists and physiotherapists during lower limb prosthetic rehabilitation, noted that clinicians lack a standardized approach to prosthetic rehabilitation. To illustrate, some prosthetists prefer removing a prosthesis to promote wound healing, whereas others believe continuing to wear it is more beneficial by encouraging blood flow.^18^ Furthermore, recent studies suggest that a limb does not need to be fully healed to begin prosthetic rehabilitation,^16^ but clear guidelines for when an open surgical site is appropriate for prosthetic use are still lacking. One prosthetist emphasized^18^ that “we have to go at the rate of the body,” noting that limbs heal and mature at different rates, further underscoring the variability in both clinical practice and patient recovery trajectories. Moreover, individuals awaiting amputation often present with multiple comorbidities that complicate their healing process. One of the most common causes of amputation is complications arising from diabetes,^19^ yet hyperglycemia can lead to vascular stiffening, microvascular dysfunction, reduced tissue oxygenation, and, consequently, impaired wound healing.^20^

The complexity of defining readiness for prosthetic rehabilitation, coupled with the lack of standardized clinical practices, suggests the need for more objective measures, such as biomarkers, to assess healing and reduce the risk of complications like revision surgeries or re-amputations. A biomarker is defined by the U.S. FDA (Food & Drug Administration) as a “defined characteristic that is measured as an indicator of normal biological processes, a pathogenic process or a response to an exposure or intervention”.^21^ Additional scholarly works have extended the FDA's definition by emphasizing the requirement for objectivity^22^ and the importance of accurate and reproducible measurements.^23^ However, to the authors' knowledge, research into the use of biomarkers for monitoring healing and facilitating early prosthetic rehabilitation post-amputation remains limited. Studies that do exist, such as those focusing on changes in tissue composition during prosthetic use,^24^ typically examine mature residual limbs, whereas early-stage residual limbs face greater risks of complications like ulceration and volume changes, which exacerbate poor socket fit.^25^ Research into these early stages is crucial for ensuring successful prosthetic rehabilitation and preventing further surgical interventions.

This raises the following research question: What biomarkers (physical, chemical, or other) are predictive, diagnostic, and/or indicative of healing of the tissues and structures found in the residual limbs of adults with amputation?

In summary, as noted by Patel et al.^26^ advances in genomics, proteomics, and molecular pathology have led to the identification of several candidate biomarkers with potential clinical value. However, progress in this area remains slow, and there is little consensus in the literature regarding the most appropriate biomarkers for assessing healing.^22^ Furthermore, to the authors' knowledge, no comprehensive review exists that synthesizes biomarkers specifically related to healing after amputation.

The most recent study examining readiness for prosthetic rehabilitation following transtibial amputation concluded that the only objective healing assessment used in the included studies was transcutaneous oxygen perfusion, a physical biomarker.^16^ The review emphasized that objective methodologies like this could quantify healing, reduce subjectivity, and promote comparative research on different enhanced recovery after surgery protocols and their effects on post-amputation healing.^16^

Existing reviews are typically narrative in nature, discussing general wound healing biomarkers without a systematic approach, further highlighting the need for a more structured review of biomarkers specific to healing in the context of amputation and primary wound healing post-surgery. To address this gap a scoping review was developed and implemented to compile the breadth of available wound healing biomarker evidence and answer the research question. The aim of the review was therefore to identify predictive, diagnostic, and/or indicative biomarkers (physical, chemical, or other) of healing of the tissues and structures found in the residual limbs of adults with amputation. To meet this aim and answer the research question, the following objectives were compiled:

**1)** Collate and synthesize the reported definitions of healing and non-healing in the literature investigating healing of the tissues and structures found in the residual limbs of adults with amputation.
**2)** Identify and collate physical biomarkers predictive, diagnostic, and/or indicative of healing repeated in sources investigating healing of the tissues and structures found in the residual limbs of adults with amputation.
**3)** Identify and collate chemical biomarkers predictive, diagnostic, and/or indicative of healing repeated in sources investigating healing of the tissues and structures found in the residual limbs of adults with amputation.
**4)** Assess the quality and levels of evidence of sources investigating healing of the tissues and structures found in the residual limbs of adults with amputation.

The term “physical” refers to biomarkers such as pH, temperature of the wound, or collagen quantity revealed through histochemical staining,^27^ whereas the term “chemical” refers to markers found in wound tissue, fluid, serum/blood, sebum, saliva, or sweat such as cytokines or matrix metalloproteinases (MMPs).

### 2: PART 1 - RATIONALE, AIMS, AND OBJECTIVES

This article (Part 1) addresses Objectives 1 and 4 and is the first in a series of three articles, each of which explores Objectives 1 to 3 in turn. Before objective measures of healing can be developed, it is essential to first clarify the current definitions of healing. The timing of prosthetic rehabilitation, for instance, is contingent upon how healing, and consequently readiness for prosthetic fitting, is defined. Likewise, effective wound management hinges on the criteria used to distinguish between a healed and an unhealed wound. However, the literature reveals a lack of consensus on the definitions of healing and non-healing wounds.^28^

While complete healing is often characterized by the “complete epithelialization” of the wound,^29–31^ this description neglects the underlying tissue layers. Where definitions of healing fall short, defining non-healing may be a useful alternative. Yet, definitions of impaired healing (commonly referred to as non-healing, chronic wound healing, or delayed healing) also exhibit significant variability. For instance, Furuyama et al.^32^ define non-healing ulcers as wounds resulting in “major amputation or death before achieving ulcer healing”, whereas another source considers a chronic wound to be one that “has not shown a 20–40% reduction in wound area after 2–4 weeks of optimal treatment”.^33^ Relying solely on temporal criteria to distinguish healing from non-healing can be problematic. For example, research has shown that while older adults may experience delayed healing, the ultimate outcome remains comparable to that of younger individuals.^34^ Additionally, Day et al.^16^ found that in their review of determinants of healing and readiness for prosthetic fitting, healing was undefined in 13 of the 15 studies reviewed. They also noted that the absence of standard healing definitions, the heterogeneity of measurable endpoints, and the inconsistent reporting of healing across studies significantly hinder the extrapolation of findings.

In light of these challenges, the following article aims to answer the research question: How are healing and non-healing defined in the literature investigating biomarkers of healing of the tissues and structures found in the residual limbs of adults with amputation?

The aim of this article is therefore to compile definitions of healing and non-healing that are provided in the literature investigating biomarkers of healing of tissues and structures found in the residual limbs of adults with amputation.

## METHODOLOGY

Given the novelty of the research question and the variable sources available on biomarkers, a scoping review was deemed the most appropriate method to meet the aims and objectives and answer the research question. The scoping review was based upon the Joanna Briggs Institute (JBI) methodology for scoping reviews^35–38^ and implemented following the Preferred Reporting Items for Systematic Reviews extension for Scoping Reviews (PRISMA-ScR) checklist and guidance.^39,40^ All results were tracked and recorded on Excel Version Number 2303 (Microsoft, Washington, USA) run on Windows 11 Version 22H2 (Microsoft, Washington, USA). A scoping review is iterative,^41^ with several steps requiring piloting; thus, the methodology presented in the following sections represents the final iterations of these processes.

### 1: INCLUSION CRITERIA

The following sections detail and rationalize the inclusion criteria of the scoping review culminating in the generation of an inclusion tool (**Table 2**) used in the first and second rounds of screening.

**Table 2:** Inclusion criteria tool applied to each source during the first (title and abstracts) and second (full text) screening processes. To pass screening one, sources required all ‘Yes’ or ‘Maybe’ answers. To pass screening two on the other hand, and be included in data extraction, sources needed ‘Yes’ responses to all inclusion criteria.

Evidence Source Details and Characteristics				
Citation				
Primary Author (Year)				
Title				
Abstract				
**Inclusion Criteria for Screening One**		**Yes**	**No**	**Maybe**
1	Does it reference biomarkers of wound healing (progression/monitoring/prediction)?			
2	Does it refer to healing of tissues found in the residuum?			
3	Is it published during or after 2017?			
**Inclusion Criteria for Screening Two**		**Yes**	**No**	
1	Does it reference biomarkers of wound healing (progression/monitoring/prediction)?			
2	Does it refer to healing of tissues found in the residuum?			
3	Does the source involve human/rat/mice participants? If it involves human participants, are they over 18 years old?			
4	Is it published during or after 2017?			
5	Is the methodology clear/repeatable?			
6	Does the study have clear ethical approval?			

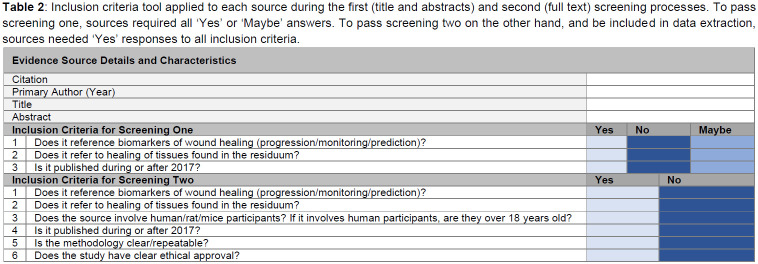

#### 1.1: Participants

To minimize the ethical considerations associated with studies involving children, given that healing in adults and children reportedly differs,^42,43^ only sources involving adult participants were included. In line with common practice in literature^44^ and UK law (the setting in which this research takes place), an adult is defined as an individual older than or equal to 18 years of age.^45^

A further inclusionary criterion was that participants must be experiencing some form of clearly described wound in tissues and structures comparable to that of an amputation residuum (**Table 3**). For example, the study by Giesen et al.^46^ meets the inclusion criteria despite focusing on risk factors, such as C-reactive protein (CRP) biomarker levels, for surgical site infections (SSI) following appendectomy. SSI is relevant as it can result in a non-healing wound.^47^ Although the infection in this case occurs at the appendix, it affects the surrounding skin and soft tissue. This tissue is biologically comparable to that found at an amputation surgical site, thereby making the findings applicable to the study's context.

**Table 3:** For clarity this table provides examples of tissues/structures found in the amputation residuum and those not.

**Examples of Tissues/Structures Found in the Residuum**
Skin, muscle and tendons, ligaments, bone, vasculature, and the peripheral nervous system.
**Examples of Tissue/Structures Not Found in the Residuum**
The central nervous system, and organs like the heart, brain, stomach, intestines, etc.

#### 1.2: Types of Sources

All the source types expressed in the following list were considered for inclusion to ensure the breadth of research was captured:

- Quantitative studies
  This includes any study design, including retrospective/prospective cohort studies, randomized controlled trials (RCTs),^48^ and in vitro, in silico, or rat/mouse studies. Note that rats/mice are considered sufficiently genetically similar to humans and are often used in biological research^49^ and will thus be included in this review. Where human participants were involved, the articles must clearly state whether ethical approval and informed consent were provided to meet the eligibility criteria.
- Qualitative studies
- Mixed studies
- Case studies
- Conference proceedings
- Dissertations and theses
- Text and opinion articles
- Letters to editors
  These may be of value given their purpose to act as a form of post-publication peer review and the platform they give researchers to share experiences with fellow readers.^50^
- Guidelines issued by national and international wound and tissue viability associations
  Examples of this include the National Institute for Health and Care Excellence (NICE) guidance on “Prontosan for treating acute and chronic wounds”^51^ and the NHS “Wound Management Clinical Practice Guidelines”.^8^

**Figure 1:**
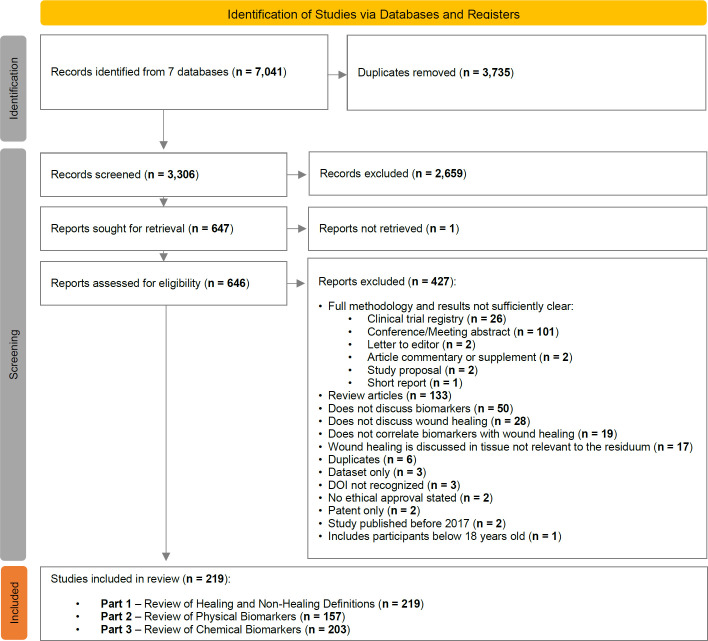
PRISMA (Preferred Reporting Items for Systematic Reviews and Meta-Analyses) flow diagram of the scoping review screening process.

However, all sources included were required to be reproducible, necessitating that their methodologies be clearly outlined. As a result, sources such as letters to editors and conference proceedings generally did not meet the inclusion criteria (**Figure 1**). Review articles were considered secondary sources and excluded.

The extensive number of sources generated during the initial searches prompted a reassessment of the inclusion criteria. Additionally, the rapid advancements in wound healing biomarkers^48^ underscored the necessity for more recent data. A recent scoping review examined prognostic factors (biomarkers) associated with ulcer healing, a common diabetic complication that can precede amputation,^52^ specifically focusing on sources published before 2017.^53^ In light of this context, it was decided to include only sources published in or after 2017, thereby ensuring the relevance and timeliness of the reviewed literature.

#### 1.3: Concept (Interventions and Outcomes)

Sources were required to explore biomarker(s) in conjunction with wound healing. A relationship between the biomarker (independent variable) and non-healing/healing (dependent variable) was required for quantitative, observational, and mixed studies. A result was considered conclusive when a statistical significance of p < 0.05 was achieved. However, measuring biomarkers can be a continuous or categorical variable, thus any sources using cut-off or dichotomizing/categorizing approaches were also included.^48^

#### 1.4: Context

Sources of any context (e.g., home, hospital, community, or academic institutions) and from any discipline (e.g., healthcare professionals or engineers) were considered to capture as much research as possible. Similarly, provided they were in the English language due to the linguistic limitations of the primary reviewer, sources from any geographical setting were considered to minimize high-income-country (HIC) and Western publication bias.^54,55^

### 2: SEARCH METHODS FOR IDENTIFICATION OF SOURCES

According to the three-step search strategy recommended by JBI, an initial search was carried out on Medline via Ovid and PubMed to locate relevant sources and determine whether or not they could contribute to increasing search terms and keywords.^56^ Following the generation of an exhaustive list of terms based on the research question, and search strategy piloting, the search terms detailed in **Table 4** were decided upon.

**Table 4:** Search terms and indexing used to generate all sources screened in the final scoping review. Note the proximity search “adj5” index applies only to Ovid databases and differs according to the database.

**Biomarker**	Biomarker*
	Marker*
	Indicator*
	Factor*
**Amputation**	Amputee*
	Amputation*
	Residuum*
	Stump*
	Limb Loss
**Wound Healing**	Wound adj5 Sensing
	Wound adj5 Sensor
	Heal/Heals/Healed/Healing
	Monitor/Monitoring
	Sensor/Sense/Sensing
	Wound adj5 Healing
	Wound adj5 Monitoring
	Wound adj5 Monitor

In a scoping review of scoping reviews, Pham et al.^57^ concluded that the most frequent limitation was the possibility of missing relevant sources, which can be attributed to database selection. To counteract this, a significant number of databases mentioned in previous scoping reviews of a similar nature^48,58,59^ were searched:

- Web of Science
- MELDINE (hosted on the Ovid platform)
- Embase (hosted on the Ovid platform)
- Scopus
- Cochrane
- PubMed
- CINAHL

All search results were exported and stored in EndNote 20 (Version 20.2.1, Clarivate, 2021) and duplicates were removed.

### 3: DATA EXTRACTION

Articles that passed both screening steps and met the eligibility criteria were then subjected to data extraction. Data (including study type, definitions of healing and non-healing, wound details, sample type, sample size, and levels and quality of evidence) was extracted in accordance with the data extraction tool (**APPENDIX A**). Despite the debate surrounding the use of quality assessment in scoping reviews,^41,60^ it was decided to systematically demonstrate that the quality of evidence collated was acceptable to enhance the validation of the results of this review. The QualSyst tool (**APPENDIX B**) proposed by the Alberta Heritage Foundation^61^ was decided upon given that it outputs a number providing a quantitative and reproducible means of identifying quality that other critical appraisal tools do not.^62^ The outputted score allows a source to be categorized as limited, adequate, good, or strong quality. Similarly, evidence levels were assessed using the JBI levels of evidence (**APPENDIX C**).

High numbers of poor-quality and low-level evidence could be considered indicative of a need for improvements in biomarker research methods.

### 4: DATA ANALYSIS AND PRESENTATION

The nature of a scoping review does not lend itself to a meta-analysis, thus it is recommended that it should instead focus on basic descriptive analysis such as frequency counts of concepts. Peter et al.^35^ further state that in some cases basic coding in a review proves useful particularly when identifying or clarifying definitions. Since the objective of this review requires the synthesis of wound healing definitions, coding is justified. To explore relationships between study types and definitions of healing and non-healings, results are subdivided into study types with frequency counts of definitions within these study types identified.

Extracted data is expressed in two primary formats. The first is a summary of the search results and selection process,^35^ including a PRISMA diagram. The second is the presentation of the data extracted from the included sources, in such a format that the research question is answered. Results are descriptively presented in paragraphs that align with the review's objectives and are diagrammatically mapped. Charts allow frequency counts to be graphically visualized. It is well-known that data visualizations enhance understanding.^63^ All charted data (including source references) are openly available in the review's dataset^64^ stored on the University of Strathclyde KnowledgeBase.

## RESULTS

### 1: OVERALL RESULTS

#### 1.1: Search Strategy Results and Included Articles

Of the 7,041 sources generated from the search strategy (**Table 5**), 3,735 were duplicates, so 3,306 titles and abstracts were screened (**Figure 1**). 2,659 sources were excluded, leaving 647 for full-text screening. After exclusion, 219 articles remained and were subjected to data extraction. Primary reasons for exclusion included review articles, unclear methodologies, no ethical approval, inaccessible texts, language constraints, irrelevant wound healing, and a lack of focus or discussion on biomarkers.

**Table 5:** Breakdown of the search strategy results for each searched database.

Database		Search Date	Number of Results	Limited to Abstracts, Titles, Keywords (specifics of the applied limit)	Limited to 2017 and after
Web of Science		06/05/2023	4,924	2,087 (abstract limit)	931
Ovid Medline		06/05/2023	2,942	2,852 (abstract limit)	1,086
Ovid Embase		06/05/2023	4,050	3,934 (abstract limit)	1,818
Scopus		06/05/2023	4,534	4,534 (title, abstract, keyword limit)	1,828
PubMed		06/05/2023	3,833	2,199 (title, abstract limit)	916
CINAHL		06/05/2023	1,014	505 (abstract limit)	245
Cochrane	Cochrane Reviews	06/05/2023	202	16 (title, abstract, keyword limit)	8
	Cochrane Protocols	06/05/2023	30	0 (title, abstract, keyword limit)	0
	Cochrane Trials	06/05/2023	318	312 (title, abstract, keyword limit)	209
				**Total References**	7,041
				**Duplicates Removed**	3,735
				**Total References to Screen**	3,306

#### 1.2: Quality and Levels of Evidence

All included evidence was quantitative with 77% of all studies^29,31,32,46,65–229^ demonstrating strong quality, and 0 studies graded with limited quality (**Table 6**). Evidence levels, on the other hand, varied more; for Prognosis 35 studies were graded level 1.b (the second highest level of evidence), and 4 (**Table 7**) were graded 5.c (the lowest level of evidence), whereas for Effectiveness, 1 and 12 studies were graded 1.b and 1.c, respectively. However, 98 studies were graded 5.c (**Table 7**).

**Table 6:** Ranking criteria for scores generated using the QualSyst quality assessment tool^61^ and numbers of included sources that obtained these rankings (NA = Not Applicable).

Quality Threshold Scores		Number (%) of Included Sources	References of Included Sources
Percentage (%) of Maximum Possible Score	Quality		
< 50%	Limited	0 (0%)	NA
≥ 50% and < 70%	Adequate	9 (4%)	231, 234, 235, 241, 253, 263, 270, 272, 274
≥ 70% and < 80%	Good	41 (19%)	230, 232, 233, 236–240, 242–252, 254–262, 264–269, 271, 273, 275–279
≥ 80%	Strong	169 (77%)	29, 31, 32, 46, 65–229

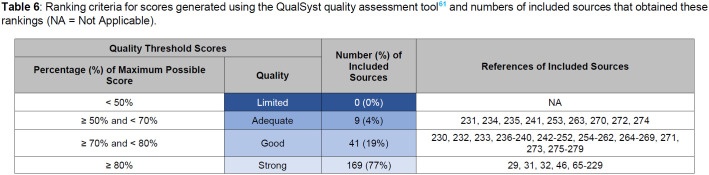

**Table 7:** Levels of evidence of the included articles in accordance with the JBI Levels of Evidence (**APPENDIX C**) (JBI = Joanna Briggs Institute; NA = Not Applicable).

Evidence Level	JBI Evidence Level Study Categories			
	Effectiveness	Diagnosis	Prognosis
1.a	0	0	0
1.b	**1** (237)	**10** (75, 85, 94, 130, 144, 158, 176, 231, 232, 234)	**35** (29, 31, 68, 74–76, 81, 83, 85, 87, 94, 97, 100, 102, 124, 132, 144, 158, 171, 175, 176, 179, 191, 195, 198, 201, 203, 206, 230–236)
1.c	**12** (103, 105, 136, 163, 212, 238–244)	NA	NA
1.d	0	NA	NA
2.a	0	0	0
2.b	0	0	0
2.c	0	NA	NA
2.d	0	NA	NA
3.a	0	0	0
3.b	**1** (159)	0	**50** (32, 46, 72, 73, 84, 88, 90, 91, 98, 104, 106, 109–113, 118, 123, 125, 126, 128, 130, 138, 139, 141–143, 148, 149, 154, 156, 161, 166, 184, 185, 187, 188, 194, 197, 211, 213, 214, 216, 220, 274–279)
3.c	**3** (77, 232, 268)	NA	NA
3.d	**3** (151, 183, 269)	NA	NA
3.e	**34** (31, 69, 75, 76, 84, 99, 102, 106, 109, 113, 114, 116, 121, 122, 125, 130, 144, 149, 153, 158, 176, 179, 185, 196, 198, 199, 201, 224, 234, 235, 270–273)	NA	NA
4.a	0	0	0
4.b	0	0	**2** (93, 151)
4.c	0	NA	NA
4.d	**1** (89)	NA	NA
5.a	0	0	0
5.b	0	0	0
5.c	**98** (65–67, 70, 71, 78–80, 82, 86, 92, 95, 96, 107, 108, 115, 117, 119, 120, 127, 129, 131, 133–135, 137, 140, 145–147, 150, 152, 155, 157, 160, 162, 164, 165, 167–170, 172–174, 177, 178, 180–182, 186, 189, 190, 192, 193, 200, 202, 204, 205, 207–210, 215, 217–219, 221–223, 225–229, 245–267)	**4** (75, 85, 94, 231)	**4** (101, 127, 131, 178)

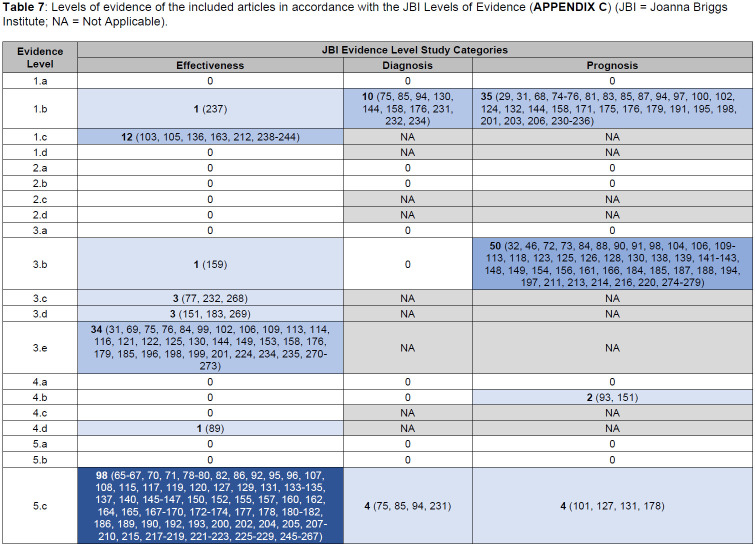

Study types additionally meant that no studies were graded for Meaning or Economic Evaluation levels of evidence. For levels of evidence, it is important to note that the total frequency counts add up to greater than 219 (the number of included articles) given that several studies were graded in more than one evidence level category; for example, often when graded for Prognosis, they were additionally graded for Effectiveness. Interestingly, 153 (70% of 219) were graded for Effectiveness, yet only 14 (6% of 219) met the criteria to be graded for Diagnosis (**Table 7**).

#### 1.3: Study Types and Settings

The most common study type was bench research, with 99 studies of this kind and only 6 case-controlled studies (**Table 8**). The most common setting research took place in was a university environment (190 studies), whereas only 1 study occurred in a governmental organization setting (**Table 9**). 66 and 35 studies were conducted in medical centers and research centers, respectively (**Table 9**). Note that the counts of settings and countries exceed 219 because 76 (35%) of the articles took place in more than one setting, and 26 (12%) of articles took place in more than one country. All included articles came from 40 countries, with 56 studies affiliated with China alone (**Table 10**). Whereas, only 7 and 3 articles were based in the UK and Ireland, respectively.

**Table 8:** Study types of all included articles.

Study Type		Number (%) of Included Sources	References of Included Sources
Bench Research		99 (45%)	65–67, 70, 71, 78–80, 82, 86, 92, 95, 96, 101, 107, 108, 115, 117, 119, 120, 127, 129, 131, 133–135, 137, 140, 145–147, 150, 152, 155, 157, 160, 162, 164, 165, 167–170, 172–174, 177, 178, 180–182, 186, 189, 190, 192, 193, 200, 202, 204, 205, 207–210, 215, 217–219, 221–223, 225–229, 245–267
Observational Study	Retrospective	52 (24%)	32, 46, 72, 73, 84, 88, 90, 91, 98, 99, 104, 106, 109–113, 118, 123, 126, 128, 130, 138, 139, 141–143, 148, 149, 154, 156, 159, 161, 166, 171, 184, 187, 188, 194, 197, 211, 213, 214, 216, 220, 224, 274–279
	Prospective	49 (22%)	29, 31, 68, 69, 74-7^7^, ^81^, ^83^, ^85^, ^87^, ^94^, ^97^, ^100^, ^102^, ^114^, ^116^, ^121^, ^122^, ^124^, ^125^, ^132^, ^144^, ^153^, ^158^, ^175^, ^176^, ^179^, ^191^, ^195^, ^196^, ^198^, ^199^, ^201^, ^203^, ^206^, ^230^-2^36^, ^268^, ^270^–^273^
Randomized Controlled Trial (RCT)		13 (6%)	103, 105, 136, 163, 212, 237–244
Case-Controlled Study		6 (3%)	89, 93, 151, 183, 185, 269

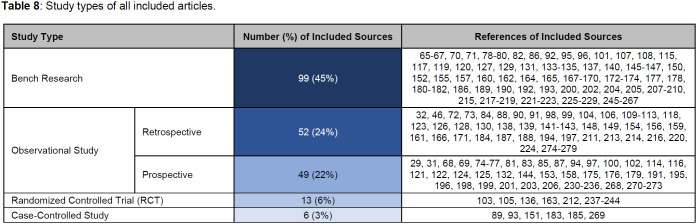

**Table 9:** Setting in which the included articles took place. Note that university includes university hospitals and some sources took place in more than one setting.

Setting	University	Medical Center	Research Center	Governmental Organization
**Number of Included Sources**	190	66	35	1
**References**	29, 31, 32, 65–73, 75–81, 83–87, 89–93, 95–108, 110–113, 115–123, 125–157, 159, 161, 163–177, 179–182, 184–195, 197, 199–210, 212–229, 231–240, 243, 244, 246–250, 252–255, 259–269, 272, 273, 275, 277, 278	22, 29, 31, 32, 46, 72, 74, 76, 78, 82, 85, 88, 90, 91, 93, 99, 100, 105, 109, 114, 123, 125, 128–130, 136, 138, 139, 148, 151, 154, 158, 167, 172, 175, 179, 194–198, 201–203, 205–207, 211, 214, 216, 228, 230, 232, 239–242, 248, 253, 255, 257, 268, 273, 274, 276, 277, 279	94, 96, 124, 141, 150, 155, 157, 160, 162, 169, 173, 174, 178, 180, 183, 184, 198, 200–202, 206, 207, 215, 228, 241, 245, 251, 255, 256, 258, 264, 267, 268, 270, 271	114

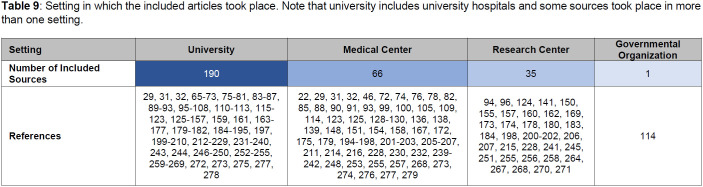

**Table 10:** Number of included sources based in each country (some studies took place in more than one country).

Country	China	USA	India	Japan	Korea	Turkey	Taiwan	UK	Italy	Germany
**No.**	**56** (70, 81, 95, 99, 102–106, 108, 115, 119, 125, 126, 128, 129, 134, 139, 140, 143, 145, 150, 168–170, 172–174, 177, 178, 196, 203, 208, 215, 217, 218, 220–229, 245, 248, 254, 255, 259, 260, 262, 267, 273, 278)	**47** (78, 83, 85, 88, 90, 92, 98, 101, 108–112, 127, 135, 137, 146, 155, 156, 160, 163, 171, 179, 181, 182, 194, 195, 199, 204–207, 213, 217, 219, 232, 246, 247, 249, 252, 257, 258, 265, 266, 268, 277, 279)	**13** (94, 96, 117, 124, 191, 198, 232, 233, 236, 241, 251, 256, 261)	**11** (29, 32, 138, 153, 158, 159, 161, 193, 211, 221, 276)	**9** (70, 120, 149, 155, 157, 166, 187, 188, 216)	**9** (68, 84, 89, 93, 97, 142, 189, 202, 275)	**7** (31, 164, 165, 167, 175, 176, 237)	**7** (24, 67, 148, 205, 243, 255, 281)	**7** (91, 122, 130, 136, 141, 214, 264)	**7** (71, 131, 174, 184, 234, 254, 278)
**Country**	Canada	Iraq	Brazil	Indonesia	France	Pakistan	Cuba	Netherlands	Ireland	Denmark
**No.**	**5** (65, 66,, 102, 123,, 134)	**5** (67, 87, 231, 250, 269)	**5** (78, 132, 180, 204, 274)	**5** (107, 151, 239, 240, 244)	**4** (86, 91, 147, 201)	**4** (75, 120, 230, 253)	**4** (183, 268, 270, 271)	**4** (46, 131, 184, 235)	**3** (67, 80, 86)	**3** (144, 197, 253)
**Country**	Saudi Arabia	Malaysia	South Africa	Iran	Singapore	Switzerland	Austria	Nigeria	Poland	Czech Republic
**No.**	**3** (190, 236, 261)	**3** (154, 210, 261)	**3** (79, 152, 186)	**3** (185, 238, 272)	**2** (209, 255)	**2** (118, 131)	**2** (131, 184)	**2** (74, 100)	**2** (77, 116)	**1** (148),
**Country**	Egypt	Israel	Thailand	Lithuania	Greece	Norway	Romania	Lebanon	Sweden	Finland
**No.**	**1** (133)	**1** (76)	**1** (192)	**1** (131)	**1** (131)	**1** (78)	**1** (89)	**1** (73)	**1** (69)	**1** (162)

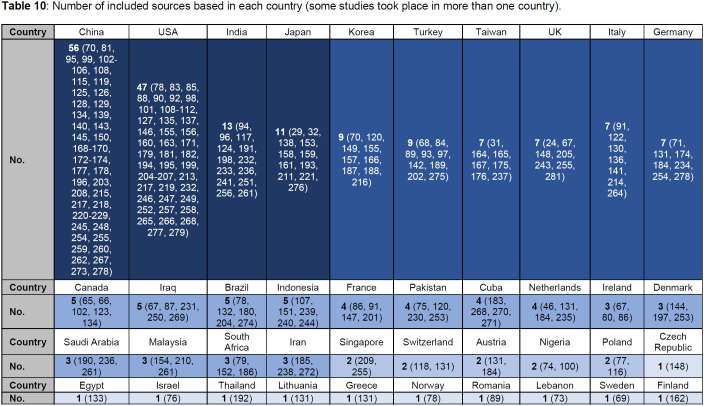

### 2: DEFINITIONS

#### 2.1: Healing Definitions

**Figure 2:**
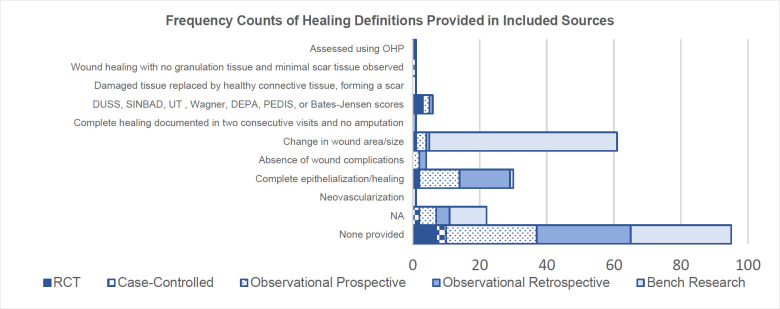
Frequency counts of healing definitions provided in all included sources, categorized by study types (OHP = hydroxyproline; DUSS = diabetic ulcer severity score; SINBAD = site, ischemia, neuropathy, bacterial infection, and depth; UT = University of Texas; DEPA = depth of ulcer, extent of bacterial colonization, phase of ulcer, and association etiology; PEDIS = perfusion, extent, depth, infection, and sensation; NA = not applicable). Note that the total frequency equates to greater than 219 (the number of total included sources) given that some included sources encapsulated two definitions in order to define healing (e.g. change in wound area and absence of wound complications).

As depicted in **Figure 2**, 43% (n = 95) of included sources (all study types) provided no definition of healing. When definitions were provided, healing was most explained by complete epithelization/healing and change in wound area/size, utilised in 14% (n = 30) and 28% (n = 61) of sources respectively. Changes in wound area were most often used in bench research studies (92% of included sources using this definition) and were commonly presented as a wound healing rate defined as follows (Equation (1)):



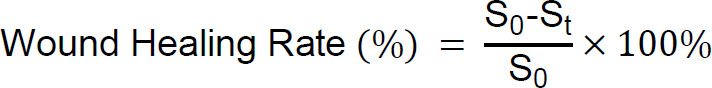



Where S_0_ is the original wound area, and S_t_ refers to the wound area at any given time after injury. Interestingly only one source^242^ incorporated biomarkers in their definition of healing, using OHP (hydroxyproline) levels as a surrogate marker of healing in their randomized control trial investigating the effects of topical negative pressure (TNP) therapy on tissue oxygenation and wound healing in vascular foot wounds.

A further 6 sources^68,76,149,163,237,244^ (all human participant studies) added a more systematic approach to the definition of healing than others through the implementation of grading systems/scales such as the Wagner Scale or the University of Texas classification system. Chen et al.,^237^ in their randomized controlled trial, defined a healed ulcer as Wagner Grade 0 (skin intact, but bony deformities lead to “foot at risk”^282^) and 1 (superficial ulcer). Lee et al.^163^ evaluated residual limb incision healing using a modified Bates-Jensen Score (mBJS) assessment tool, scoring the following criteria from 1 to 5: amputation skin color, epithelization, amount of exudate, and the presence and volume of eschar. Higher scores therefore indicate worse healing. In fact, Jeon et al.,^149^ in their observational retrospective study, employed and compared five classification systems for diabetic foot ulcers (Meggitt-Wagner classification; SINBAD [site, ischemia, neuropathy, bacterial infection, and depth] score; DEPA [depth of ulcer, extent of bacterial colonization, phase of ulcer, and association etiology] scoring system; UT [University of Texas] diabetic wound classification; DUSS [diabetic ulcer severity score]) to identify the “gold standard” prognostic classification system or optimum prediction tool for amputation.

#### 2.2: Non-Healing Definitions

**Figure 3:**
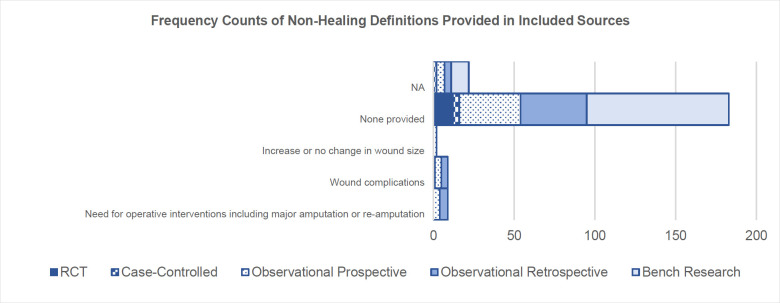
Frequency counts of non-healing definitions provided in all included sources, categorized by study types (NA = not applicable). Note that the total frequency equates to greater than 219 (the number of total included sources) given that some sources encapsulated two definitions (for example MacDonald et al.^83^ used no change in wound size and presence of wound complications) in order to define non-healing.

Over 80% (n = 183) of included sources provided no definition of impaired or non-healing wounds (**Figure 3**). In the limited sources (all were human participant studies) where a definition was stated, the identification of wound healing complications (**Table 11**), increase or no change in wound size, or the need for operative interventions, explained non-healing in 4% (n = 9),^83,109,112,163,179,195,206,216,276^ 1% (n = 2),^83,158^ and 4% (n = 9)^32,109,118,179,194,195,206,216, 273^ of sources respectively. In none of the definitions were biomarkers used. Wound complications were defined differently depending on the source, as compiled in **Table 11**. Of the 9 sources using wound complications to define non-healing, 67% (n = 6) explored healing in relation to the amputation surgical site.^109,112,163,206,216,267^

**Table 11:** Wound healing complications stated in non-healing definitions coded for ‘wound complications’ (RCT = randomized controlled trial; CLTI = critical limb threatening ischemia; SSI = surgical site infection).

Source	Study Type	Wound Type	Non-Healing Definition Wound Complications
Lee et al. (163)	RCT	Amputation	Signs such as erythema, drainage, infection, incision breakdown, skin/fat necrosis, and/or tissue eschar.
Majumdar et al. (179)	Observational Prospective	Surgical Site After Lower Extremity Revascularization	Need for operative interventions for SSI or dehiscence, or new ulcerative wound or bypass graft infection.
Nystrom et al. (195)	Observational Prospective	Surgical Site After Lower Extremity Soft Tissue Sarcoma Excision	Any wound-related issue (necrosis, dehiscence, infection, seroma) treated by a return to the operating room, initiation of oral or intravenous (IV) antibiotics, intervention for seroma including aspiration, or prolonged wound packing or dressing changes greater than 120 days.
Squiers et al. (206)	Observational Prospective	Lower Limb Amputation	Development of necrosis; development of infection, including gangrene or abscess; ulceration occurring within or adjacent to the surgical wound; disruption or dehiscence of suture line; drainage or exudate expressed from the suture line; evidence of inflammatory response including swelling, cellulitis, or skin discoloration; hematoma formation; revision of the amputation to a more proximal level.
MacDonald et al. (83)	Observational Prospective	Diabetic Foot Ulcer	Pain, erythema, oedema, heat, purulent exudate, serous exudate with concurrent inflammation, delayed healing, discoloration of granulation tissue, friable granulation tissue, pocketing at the base of the wound, foul odor, and wound breakdown.
Adams et al. (109)	Observational Retrospective	Transmetatarsal Amputation	(1) revision of the amputation, defined as a return to the operating room for any reason; (2) postoperative infection, defined as any superficial or deep infection requiring oral antibiotics, admission to the hospital for intravenous antibiotics, and/or an unplanned return to the operating room; (3) chronic residual limb ulceration, defined as a non-healing wound at the surgical site requiring >4 weeks of wound care; (4) calcaneal gait, defined as any increased pressure at the plantar heel resulting in a pressure sore; (5) residual limb deformity, defined as a nonplantigrade foot; and (6) residual limb infarction, defined as ischemia or necrosis of the incision site.
Alfawaz et al. (112)	Observational Retrospective	Below-Knee Amputation	Separation or necrosis of skin, flap necrosis, or dry ischemic eschar formation.
Morisaki et al. (276)	Observational Retrospective	Above or Below-Knee Amputation	Surgical site infection or wound dehiscence.
Woo et al. (216)	Observational Retrospective	CLTI Patient Ulcer or Amputation Surgical Site	Wounds requiring regular dressing and antibiotic treatment or surgical wound revision and additional surgery.

In addition to major amputation, Furuyama et al.^32^ further defined ulcer non-healing in patients with critical limb ischemia by amputation or death. Contrastingly Kimura et al.^158^ defined worsened foot wounds only as wounds that had increased in size without amputation, with participants resulting in minor or major amputation, or all-cause death in the one-year study period being classified separately.

In 10% (n = 22) of all included sources^80,85,86,88,94–96,106,121,127,144,145,169,178,183–185,205,207,234,253,279^ both healing and non-healing definitions were considered not applicable given the content of the research. Laiva et al.,^80^ for example, explore the expression of pro-angiogenic factors (characteristic of wound healing) in human diabetic adipose-derived stem cells cultured on collagen scaffolds. Although this is investigating aspects of ulcer healing and is therefore relevant to the scoping review research question, it focuses on a specific cellular aspect of non-healing diabetic foot ulcers (DFUs), rather than in vivo whole ulcer healing (where several tissues and cells are involved).

## DISCUSSION

This scoping review aimed to compile definitions of healing and non-healing found in the literature investigating biomarkers of healing in the tissues and structures of residual limbs of adults with amputation. The findings indicate a significant lack of standardized definitions of healing within the literature, with only one source^242^ incorporating biomarkers (an objective measure rather than a subjective one) to define healing. Systematic methods for quantifying healing, such as pre-defined grading systems or scales like the Wagner Scale, were utilized in only 2% of the studies included. Moreover, these tools are generally designed for the assessment of open wound healing rather than surgical site healing. Similarly, definitions of non-healing were either absent or inconsistently characterized by varying descriptions of wound complications.

The review highlights a broader lack of consensus and standardization in defining both healing and non-healing, as current definitions are often superficial and predominantly based on visual and size-based assessments. These approaches fail to consider deeper tissue healing and mechanical properties essential for functionality, particularly in the context of prosthesis use. There is a critical need for more comprehensive, multidimensional definitions that incorporate objective measures like biomarkers and mechanical assessments, along with social and psychological evaluations, to more accurately reflect the complex nature of healing to guide future research and clinical practice more effectively.

### 1: OVERALL SEARCH RESULTS

No set number of articles should or should not be included in a review,^283^ and the number of included articles comes down to the search strategy and inclusion criteria. In this review, an arguably large number of articles (219) met the inclusion criteria, whereas in the similar work by Day et al.^16^ on determinants of healing and readiness for prosthetic fitting after transtibial amputation, 2,067 articles met the search strategy yet only 20 passed both screening stages. This difference is likely due to their inclusion criteria of transtibial amputation; in this review with the knowledge that the literature on healing on amputation specifically is low, the research question was expanded to wound healing of tissues like that of the lower limb residuum, thus broadening the number of search results.

Of the 195 countries in the world, research from 40 of these countries was included in this review, several of which were LMICs (low-to-middle-income countries such as Cuba, Egypt, China, Malaysia, Nigeria, Thailand, and Pakistan).^284^ Such global research allows us to expand findings across populations, regions, and cultures,^285^ reduces Western publication biases, and is critical in overcoming global health challenges^286^ like wound healing. It can be argued that the high number of countries from which research in this review originates highlights the global burden of wound healing. This is reinforced by the reported average of $2.8 billion spent globally on wound healing in 2014.^287^ Guest et al.^288^ concluded that in the UK alone, between 2017 and 2018, the cost to the NHS per healed wound ranged from £698 to £3,998 per patient, and that of an unhealed wound ranged from £1,719 to £5,976 per patient.

Interestingly, Tricco et al.,^289^ in their scoping review of scoping review methodologies, revealed that 423 (86%) of the articles that met their inclusion criteria did not use a quality appraisal tool in their scoping review. However, it is well reported that critical (or quality) appraisal tools are a justifiable addition to a review to systematically assess the credibility of the research on which the results of the scoping review are then based.^290^ On the other hand, Tod et al.^290^ further note that quality checklists, like the QualSyst tool, lack evidence to support their use; thus, quality assessment acts as an outcome measure, not an exclusionary criterion in this review.

As detailed in the results section (*Quality and Levels of Evidence*) the high number of Effectiveness 5.c levels of evidence can be attributed to the 99 bench research studies (almost 50% of the included articles) that were included in data extraction. Of the 99 studies, 81 were rat or mouse studies, reinforcing the justification of bench research receiving the lowest level of evidence following the JBI levels of evidence. The lower number of higher-level evidence studies can be explained by the cost of studies such as RCTs (estimated to cost anywhere in the range of $43 to 103,254 per patient),^291^ and the common lag (as long as 17 years) in translating scientific discoveries (produced through bench research) into patient studies and thus patient benefit.^292^

### 2: DEFINITIONS OF HEALING AND NON-HEALING

In their review of complete wound closure definitions, Gould and Li^28^ recorded that complete/full/100% (re)epithe-lialization or closure was the most common definition of healing. The same was noted here, of the 102 sources (47% of all included sources) that provided definitions of healing, 30 were regarding epithelialization, and 61 were defined by changes in wound size/area. However, this assessment is limited in its applicability, particularly for surgical sites, such as amputation, which do not involve open wounds. The reliance on wound size to indicate healing, particularly through methods like measuring with disposable tapes,^293^ is problematic due to poor inter-rater and intra-rater reliability, its time-consuming nature, and issues inaccuracy.^294–296^ Importantly, this focus on epithelialization alone does not capture the entirety of the healing process, as the proliferation phase, in which epithelialization occurs, is only one of four phases of wound healing. Re-epithelialization occurs in the third phase, the proliferation phase (which takes place days to weeks after injury), where granulation tissue is formed, the epidermis is regenerated and neovascularization occurs.^2^ This phase is then followed by the fourth and final phase which occurs weeks to months after injury, remodeling, characterized by vascular maturation and regression, collagen remodeling, and the point at which a wound reaches its maximum strength and ultimate endpoint.^2,3^ In cutaneous tissue for example this final phase is marked by a collagenous scar. Therefore, it can be argued epithelialization suggests healing but does not indicate a fully healed wound. Particularly in the case of an amputation where the suture line may appear healed after re-epithelization has occurred, but the final phase of healing is still taking place below the skin and is likely heavily influenced by prosthetic use (and its subsequent mechanical loading).^24^ For example, Bramley et al.^8^ conducted a study investigating changes in tissue composition and load response on 10 individuals with unilateral transtibial amputations, who had undergone the procedure between 1 and 35 years prior to the study (mean of 7.5 years) and were therefore classified as having mature residual limbs.^25^ The findings indicated a higher presence of adipose tissue infiltrating the muscle in residual limbs compared to intact contralateral limbs, suggesting muscle atrophy and adaptation post-amputation.^8^ Furthermore, intramuscular adipose content was found to correlate negatively with daily prosthetic socket use, reinforcing the idea that prosthetic use influences tissue composition in mature residual limbs, and likely has an even greater impact on early healing residual limbs. Therefore, a more comprehensive approach to defining healing should consider the deeper, ongoing processes beyond surface closure.

Definitions of non-healing were more infrequent and when provided were complex, typically focusing on the identification of complications or deviations from normal healing. One possible reason for the limited reporting of non-healing definitions is the assumption by researchers that by defining healing, non-healing is implicitly understood as the opposite. Or perhaps the challenge of clearly defining non-healing is a symptom of the complexity of a chronic wound, its causes, and the variety of systemic (for example age,^297^, sex hormones,^298^ alcoholism,^299^ smoking,^300^ and nutrition^301^) and local (for example infection,^302^ oxygenation,^303^ and venous sufficiency^304^) factors that impact healing.^4^ It is noteworthy that among the sources surveyed, studies focusing on amputation surgical sites were the primary providers of definitions for non-healing wound complications (6 of 9 included sources ^83,109,112,163,179,195,206,216,276^). This trend may arise from the fact that traditional definitions of open wound healing, like epithelization or wound site evaluation, do not readily apply to closed surgical site wound types. Furthermore, individuals undergoing amputation often present with multiple comorbidities, such as diabetes and peripheral vascular diseases,^19^ and systemic factors for non-healing, such as smoking and alcohol use,^305,306^ which can negatively impact the healing process.^20,299,300,307^ For instance, Lind et al.^306^ retrospectively examined the impact of smoking on post-operative complications in 137 patients who had undergone primary above-knee or below-knee amputations, 44 of whom were cigarette smokers. The study found that smokers had a 2.5 times higher risk of infection and re-amputation compared to non-smokers, concluding that abstaining from smoking during the post-operative healing phase is critical, as nicotine compromises cutaneous blood flow velocity and increases the risk of microthrombus formation.^306^ It can also be argued that healing complications such as infection or excessive oedema are primary barriers to prosthetic readiness, and thus of greater concern to prosthetists and rehabilitation professionals than indicators of healthy healing. Churilov et al.,^308^ for example, observed that the use of rigid dressings post-transtibial amputation, hypothesized to reduce swelling and promote healing, significantly shortened the time from amputation to casting or fitting of the first prosthesis, compared to traditional soft elastic dressings. In summary, identifying abnormal healing processes, particularly in the context of amputation, requires a more comprehensive approach than surface level visual assessments. A standardized system, tailored to specific wound types, would improve the clarity and consistency of healing and non-healing definitions.

A biomarker, however, would allow both healing and non-healing to be defined and monitored objectively and quantitatively. Unfortunately, only one included source^242^ considered a biomarker in their definition of wound healing stating that they were to “demonstrate the effects of TNP on the healing of acute wounds of the foot by measuring the change in wound volume and collagen deposition”, enlisting OHP as a well-reported surrogate marker of collagen.^242^ In addition to deposition during the proliferative phase of healing, collagen, a key component of the extracellular matrix, induces platelet activation and aggregation in response to injury (phase one of healing), promotes fibroblast recruitment in the inflammatory stage, and influences remodeling of the extracellular matrix (ECM) increasing the tensile strength of the wound in the final remodeling/maturation phase.^309^ Chiang et al.^242^ further reported that wound volume reduction from day 0 to day 14 of treatment was not significant (44.2% TNP vs 20.9% control; p = 0.15) suggesting that TNP did not expedite wound healing as expected. Similarly, the degree of collagen deposition (OHP content in tissue samples was expressed in micrograms of collagen per milligram of granulation tissue) on day 14 was also not significant between control and TNP-treated groups (58% TNP vs 94.5% control; p = 0.32).^242^ In terms of absolute values, the TNP group noted a larger reduction in wound size, but the control group observed a greater increase in collagen deposition. This reinforces the notion that there is more to the healing process than simply the dimensions of the open wound. Thus, biomarkers could provide a more nuanced and objective means of tracking both healing and non-healing across all wound types, including surgical sites. Biomarkers have also been demonstrated in osteoarthritis research to indicate responses to loading tasks, providing valuable insights into joint health and predicting structural changes.^310^ This knowledge could be applied to monitoring the health of the residual limb, which undergoes adaptation during healing and early prosthetic use. For instance, in a posterior flap below-knee amputation, the gastrocnemius muscle forms a significant part of the muscle bulk covering the residual tibia. During prosthetic use, this muscle is subjected to forces in directions it would not experience in an intact limb, necessitating adaptation in response to these forces.

Although not utilizing biomarkers, definitions in 6 sources^68,76,149,163,237,244^ appeared to adopt a more systematic approach to assessing healing through the use of scales and classification systems such as the Wagner (or Meggitt-Wagner) system. Bar the modified Bates-Jensen (mBJS) adopted by Lee et al.,^163^ the classifications used apply only to diabetic open wounds or ulcers and again rely only on visual/surface level assessment external to the wound, limiting their relevance to surgical wounds. Diabetic foot ulcers (DFUs) account for much of the research on wound healing due to their global burden, with 80% of lower extremity amputations (LEAs) linked to DFUs.^311^ However, overemphasizing DFUs risks overlooking the specific needs of amputation sites, which require different criteria for assessing healing. The aforementioned mBJS which evaluates necrotic tissue topes, necrotic tissue volume, exudate type, skin color surrounding the wound, and epithelialization on a scale of 1 (best healing) to 5 (worst healing),^312^ although designed specifically for residuum healing assessment, is also limited to observer interpretation of the surgical site. Though not used in included sources, further surgical site healing classifications exist like the Centers for Disease Control (CDC) Surgical Wound Classification (SWC)^313^ and the Surgical Wound Assessment Tool (SWAT),^314^ but again they incorporate only a variety of subjective observations and are focused primarily on the identification of surgical site infections only. Despite being more holistic tools, these classifications still provide only subjective indicators of what is occurring under the skin and are therefore limited in truly assessing deep tissue healing; limitations that could be solved with more objective measures like biomarkers.

Interestingly, all the definitions of healing and non-healing focus purely on the physical components of wound healing. The optimal healing environments (OHE) framework however suggests that patient healing is best supported by addressing not just the physical, but the social, psychological, spiritual, and behavioral components of healthcare.^315^ Doering et al.,^316^ for example, observed that in 72 patients with bypass surgery, those with higher depressive symptom scores (indicating more symptoms) reported poorer emotional recovery (p < 0.001) and poorer physical recovery (p = 0.007) and achieved shorter walking distances (p <0.001) than did patients with lower scores (indicating fewer symptoms). Furthermore, by 6 weeks after discharge, infections and impaired wound healing were more common among patients with higher depressive symptom scores (46%) than among patients with lower scores (19%, p = 0.03).^316^ Similarly, it is well known that amputation has psychological effects, with one review revealing that across 12 studies the prevalence of psychiatric disorders among amputees in India is in the range of 32% to 84%, including depression rates of 10.4% to 63% of the studied population, posttraumatic stress disorder rates of 3.3% to 56.3%, and phantom limb phenomenon rates of 14% to 92%.^317^ These symptoms of anxiety and depression reportedly do improve over time,^317,318^ yet no definitions of amputation healing detailed in this scoping review alluded to anything other than the physicality of the surgical site. Perhaps in the future, more effort should be made to consider more than the physical aspects when defining healing, providing a more holistic definition of healing.^315,319,320^ An amputation is a life-changing event; with more objective and well-explained definitions of healing individuals with amputations may feel more comfortable about their surgical site healing journey which is currently limited by biases introduced by the timing of clinician visits and subjective surface level wound examination only.^16,321^

Overall, the lack of provided definitions, irrespective of evidence level, wound type, or study type, raises concerns. For example, 13 included sources were RCTs (**Table 8**), the highest level of evidence, yet of these only 6 and 1 provided healing163^163,237,238,241,242,244^ and non-healing^163^ definitions respectively. Despite investigating healing, or an aspect of it, by not defining healing and non-healing the methodological rigor of the study is reduced by not providing a clear endpoint definition, and the belief that assessing wound healing is a purely visual process is perpetuated. As noted in previous studies^16,321^ the gap in the literature on healing definitions, particularly for amputation sites, remains unaddressed for over 20 years, despite its significance to patient outcomes. A shift toward more objective, comprehensive measures, incorporating biomarkers, psychological factors, and standardized definitions, would greatly enhance the study of wound healing in clinical settings.

To develop a tailored and relevant scale for assessing wound healing in the context of residual limbs post-amputation, the authors believe the following considerations should be made to ensure that it is comprehensive, objective, and clinically useful:

1. Incorporate all four phases of healing, capturing both surface level and deeper tissue healing processes.
2. Incorporate objective measures like biomarkers:
  - This will require identifying the most appropriate biomarkers for assessing post-amputation healing, potentially through a scoping review or bench research. For example, determining which biomarkers best assess the residual limb's capacity to withstand prosthetic fitting could include indicators of healing complications like infection, inflammation, cell death, or response to mechanical loading. Song et al.^322^ identified that inflammatory markers such as white blood cell count, serum C-reactive protein levels, and erythrocyte sedimentation rate were significantly correlated with wound healing rates in diabetic patients. Additionally, thresholds or cut-off values for these biomarkers should be established to differentiate between healing and non-healing. For instance, a transcutaneous oxygen pressure (TcPO_2_) value below 40 mmHg has been associated with a 24% increased risk of healing complications in lower limb amputations, compared to values above 40 mmHg.^323^
  - Techniques to quantify these biomarkers must be developed or adapted. This could involve quantitative imaging techniques such as ultrasound, which has been used to observe deeper tissue changes and predict the prognosis of pressure injuries,^324^ or innovative tools like wearable smart bandages capable of sensing wound pH, temperature, bioimpedance, glucose, oxygen, proteins, or uric acid in real-time.^325^
3. Include subjective and psychosocial factors:
  - Psychological markers, such as anxiety, depression, and body image perception, should be addressed, as they influence overall recovery.^316,317^ A number of existing validated tools used in the lower limb amputee population are available such as the Hospital Anxiety and Depression Scale.^326,327^
  - Patient-reported outcomes (PROs), such as the Visual Analogue Scale (VAS) for pain^328^ and the Prosthetic Limb Users Survey of Mobility (PLUS-M),^329^ can capture the patient's perspective on pain, mobility, and comfort, offering deeper insights into functional recovery and prosthetic readiness. Research should explore which outcome measures most effectively reflect prosthetic readiness, perhaps through a pilot study investigating the effectiveness of different measurement tools in monitoring post-amputation healing. The COMET (Core Outcome Measures in Effectiveness Trials) initiative provides a list of key outcome measures for studies of people undergoing major lower limb amputation for complications of peripheral vascular disease, including death, quality of life, mobility, and social integration/independence,^330^ which can serve as a foundation to be built upon with more objective measures like biomarkers.

A multi-tiered grading system should be created, where each grade corresponds to specific milestones in the healing process, defined by clear criteria. For instance, Gethin et al.^331^ conducted a scoping review and identified normal wound bed temperature in chronic wounds as being between 30.2°C and 33.0°C. For each criterion, clear healing versus non-healing indicators should be established, distinguishing between successful healing and complications such as infection or excessive oedema. This will require participant research to identify objective indicators of both healthy (e.g., a decrease in temperature and pH^332^) and unhealthy (e.g., an increase in inflammatory markers^333^) healing processes. The classification system must undergo rigorous pilot testing and validation. This includes:

- Reliability testing, ensuring high inter-rater and intra-rater reliability through testing in diverse clinical settings.
- Construct validity testing, comparing the system against known standards to confirm its accuracy.
- Patient-centered validation to ensure that users' opinions are incorporated during all stages of development to guarantee the scale addresses meaningful aspects of their recovery journey.^334^

In the future, automation and streamlined assessment processes could be explored, for example, potentially incorporating wearable sensors for remote monitoring of residual limb health during healing. This could enhance the scale's practicality and accessibility. It is also essential that the scale should undergo longitudinal tracking, allowing for continuous feedback and refinement. Regular updates or revisions should be made based on new research or clinical findings to reflect the evolving understanding of wound healing. By incorporating these elements, the scale will be robust, adaptable, and capable of providing both clinicians and patients with valuable insights into the healing process and readiness for prosthetic use.

### 3: METHODOLOGICAL DISCUSSION

#### 3.1: Methodological Strengths

A scoping review appears to be the most suitable approach to answering the research question due to its ability to comprehensively explore the extensive and unclear literature on impaired and healthy wound healing biomarkers and definitions, without restrictions on source types. In contrast, a systematic review would necessitate a more narrowly defined research question.

A key strength of this review is simply the significance of the conclusions drawn. By highlighting both the lack of healing definitions and the limitations within provided definitions, this systematically implemented review reinforces the need for further research into objective measures to quantify healing. The sooner we can reach a consensus on the most appropriate definition of healing (both cutaneous and subcutaneous), the sooner we can identify or predict a healing/non-healing wound, and the sooner it can be prevented or treated.^22^

#### 3.2: Methodological Limitations

Despite the implementation of an exhaustive search strategy, there is always a likelihood that some sources may have been missed. Therefore, it is important to remember that the results of the scoping review will guide future work; they will not influence healthcare policy, for example.

A further limitation is the current lack of a second reviewer contradicting the JBI's recommendation for a minimum of two reviewers to validate results, remove bias,^35^ and increase the number of relevant articles included in a review.^335^ However, given the nature of the authors' resource constraints, only the primary author of this study could act as the reviewer, and the supervisory team acted as a verifier. Again, it is important to consider the purpose of the review;^336^ for example, is it impacting policy? If so, then it is particularly pertinent to ensure the methodology and the inclusion/exclusion criteria are rigorously justified and piloted. The review reported here, although thorough, is not intended to directly impact policy, and the lack of a second reviewer is perhaps more justified. Furthermore, this is not too dissimilar to peer-reviewed and published scoping reviews, with the work of Tricco et al.^289^ (a scoping review of scoping reviews) revealing that only 34% of reviewed scoping reviews included two or more independent reviewers. Yet simply introducing a standardized data extraction form, as the review reported here did, can minimize bias.^35^

In the future, it would be beneficial to consider using multi-lingual reviewers given that only sources in or translated into the English language could be investigated, potentially increasing Western publication biases.^54,55^ The choice was made to refrain from utilizing online translation software due to the potential risk of semantic loss. Van Nes et al.^337^ for example recommend the use of a professional translator given that translation is an interpretative act in which meaning can be lost. However, this option is costly and falls beyond the scope of the research supporting this manuscript. Although including all study types ensures more relevant sources are captured, the inclusion of rodent studies and mathematical models can be questioned.

In review studies, a balance between high precision (narrow) and high recall (broad) searches is necessary to ensure sufficient studies are captured by the search whilst the time required to screen all included articles is feasible.^60^ As such, this step was deemed unfeasible; assuming 300 articles were included with 100 references each, a further 30,000 articles would need to be screened; this was considered not an option given the limited project timescale of the primary author.

Please note that a registered and published protocol for this review is not available, which may influence the consistency and transparency of the review process.

### 4: ETHICAL CONSIDERATIONS

The use of grey literature in reviews is a contentious topic. Searching for it can be time-consuming and it lacks the validation peer-reviewed literature can provide; however, it can reduce publication bias given that it provides data that is not found in commercially published articles.^338^ Thus, this review did aim to include grey literature however all that was generated during the searches did not meet the inclusion criteria; often failing to provide a sufficiently clear methodology and clear ethical approval.

RCTs are considered the highest level of evidence,^280^ however, they are expensive, and funding is limited. They are often industry-funded and therefore more likely to report a statistically significant positive outcome than studies without industry funding.^339^ Thus, evidence level has not been used as an exclusionary criterion in this scoping review. It was a requirement, however, that all included articles, where applicable, clearly stated ethical approval and sought informed consent when human participants were involved.

## CONCLUSION

The aim of this review was to compile definitions of healing and non-healing provided in the literature investigating biomarkers of healing of the tissues and structures found in the residual limbs of adults. Wound healing was predominantly characterized by epithelization and wound closure, including healing rates, or left undefined. Non-healing was often poorly explained, typically assessed by the need for operative intervention including re-amputation or signs of impaired healing when defined. This review highlights shortcomings in current definitions of healing and non-healing, which are frequently absent or based on superficial assessments influenced by clinician perspectives. These definitions mistakenly equate wound appearance and size with healing at deeper tissue levels, neglecting to account for the mechanical properties of the tissue that are critical, particularly in tissue subjected to loading during lower limb prosthesis use. This underscores the need for a more comprehensive approach to wound healing assessment, integrating biomarkers and potentially incorporating social and psychological evaluations, as a patient's environment significantly impacts their healing process. Before we can enhance wound management both before and after amputation and expedite the return to daily activities, it is essential to establish a clear consensus on what defines the healing and non-healing processes.

## DECLARATION OF CONFLICTING INTERESTS

The author has no conflicts of interest to declare.

## AUTHORS CONTRIBUTION

- **Hannelore Williams-Reid**: the primary author of the manuscript, undertook the scoping review and prepared the final manuscript as part of a 4-year PhD program.
- **Arjan Buis:** the primary PhD supervisor, assisted in developing the scoping review methodology and preparing the manuscript for publication.
- **Anton Johannesson**: the secondary PhD supervisor, assisted in developing the scoping review methodology and preparing the manuscript for publication.

All authors have read and approved the final version of the manuscript.

## SOURCES OF SUPPORT

The PhD project under which this scoping review/manuscript falls is funded by the UKRI EPSRC as part of the Centre of Doctoral Training (CDT) in Prosthetics and Orthotics (P&O) (studentship 2755854 “Wound management and early prosthetic rehabilitation” within project EP/S02249X/1) and by Össur.

## Abbreviations & Acronyms:

**Abbreviation & Acronym: Definition**
CDC: Centers for Disease Control
CLTI: Critical Limb Threatening Ischemia
COMET: Core Outcome Measures in Effectiveness Trials
CRP: C-Reactive Protein
DEPA: Depth of ulcer, Extent of bacterial colonization, Phase of ulcer, and Association etiology
DFU: Diabetic Foot Ulcer
DTI: Deep Tissue Injury
DUSS: Diabetic Ulcer Severity Score
ECM: Extracellular Matrix
FDA: Food & Drug Administration
HIC: High Income Country
IV: Intravenous
JBI: Joanna Briggs Institute
LEAs: Lower Extremity Amputation
LMICs: Low to Middle Income Countries
mBJS: Modified Bates-Jensen Score
MMPs: Matrix Metalloproteinases
n: Number
NA: Not Applicable
NHS: National Health Service
NICE: National Institute for Health and Care Excellence
OHE: Optimal Healing Framework
OHP: Hydroxyproline
PEDIS: Perfusion, Extent, Depth, Infection, and Sensation
PLUS-M: Prosthetic Limb Users Survey of Mobility
PRISMA-ScR: Preferred Reporting Items for Systematic Review and Meta-Analyses for Scoping Reviews
PROs: Patient Reported Outcomes
RCTs: Randomized Controlled Trials
SINBAD: Site, Ischemia, Neuropathy, Bacterial Infection, and Depth
SSI: Surgical Site Infection
SWAT: Surgical Wound Assessment Tool
SWC: Surgical Wound Classification
TcPO_2_: Transcutaneous Oxygen Pressure
TNP: Topical Negative Pressure
UK: United Kingdom
USA or US: United States of America
UT: University of Texas
VAS: Visual Analogue Scale

## Appendix

### APPENDIX A:

**Table A.1:** (adapted from References 48 and 53): Data extraction tool used to extract data from all sources that passed both screening steps. NAST refers to data extraction categories that may not be applicable to all source types.

Data to be Extracted	Clarification of Data Extraction Category
**Scoping Review Details**	
Scoping Review Title	Wound Management, Healing, and Early Prosthetic Rehabilitation: Part 1 - A Scoping Review of Healing and Non-Healing Definitions
Review Objectives	Summarized in Manuscript Section 1 (Introduction)
Review Questions	
**Evidence Source Details and Characteristics**	
Citation Details	Full Harvard APA 7th edition citation for the source including source URL.
Study Type	For example, an observational retrospective or case-controlled study.
Country	The geographical location where the source was generated.
Setting	For example, a hospital/medical Center, university, or research center.
One Sentence Summary	Summary of the study in one sentence.
**Details/Results Extracted from the Sources of Evidence**	
Participant Characteristics	For example, age range, gender, and comorbidities. Includes control group characteristics also.
Sample Type and Size	Refers to the number and type of participants investigated in the source (NAST).
Wound Details	This includes any details about the wound type, such as classification, average size, and burn or ulcer.
Follow-up Time	Refers to the time between or after reported outcome measures (NAST).
Definition of Healing	Definitions and terms are given for healthy (non-impaired) healing.
Definition on Non-Healing	Definitions and terms are given for unhealthy (impaired) healing.
Chemical Biomarkers Discussed	All chemical biomarkers discussed/measured in the source must be recorded here. See Manuscript Introduction for a definition of chemical.
Physical Biomarkers Discussed	All physical biomarkers discussed/measured in the source must be recorded here. See Manuscript Introduction for a definition of physical.
Other Biomarkers Discussed	All remaining biomarkers that do not fall into the chemical or physical category must be recorded here.
Biomarker Measurement Techniques	Summary of the discussed/used/described biomarker measurement techniques used in the source.
Outcome Measures	Reported outcome measures (aside from aforementioned biomarkers); for example, 3-year mortality may be the primary outcome measure.
Significant Results	Results of importance as judged by the reviewers.
Limitations	Key limitations of the biomarkers, biomarker quantification technologies, or methodologies that are explicitly mentioned in the source.
Level of Evidence	Level of evidence according to the JBI classification (Reference 280) (see Appendix C).
Quality of Evidence	Quality of evidence score generated using the QualSyst tool (Reference 61) (Appendix B).

### APPENDIX B:

**Table B.1:** (Reference 61): QualSyst tool checklist for assessing the quality of quantitative studies. Note that NA is not an option for Criteria 1, 2, 4, 13 and 14. Each response is assigned a point score depending on how well it meets the criteria (“yes” = 2 points, “partial” = 1 points, and “no” = 0 points). Items not applicable to a certain study design are labelled as NA and excluded from the total score. A summary score is calculated by summing the total score and dividing by the possible score (the possible score is the maximum score (28 points) minus the number of “NA” responses multiplied by 2).

Criteria		Yes (2)	Partial (1)	No (0)	NA
1	Question/objective sufficiently described?				
2	Study design evident and appropriate?				
3	Method of subject/comparison group selection or source of information/input variables described and appropriate?				
4	Subject (and comparison group, if applicable) characteristics sufficiently described?				
5	If interventional and random allocation was possible, was it described?				
6	If interventional and blinding of investigators was possible, was it reported?				
7	If interventional and blinding of subjects was possible, was it reported?				
8	Outcome and (if applicable) exposure measure(s) well defined and robust to measurement/misclassification bias? Means of assessment reported?				
9	Sample size appropriate?				
10	Analytic methods described/justified and appropriate?				
11	Some estimate of variance is reported for the main results?				
12	Controlled for confounding?				
13	Results reported in sufficient detail?				
14	Conclusions supported by the results?				

**Table B.2:** (Reference 340): Ranking criteria for scores generated using the QualSyst quality assessment tool. The tool consists of a quantitative study checklist with 14 criteria. Each criterion can be scored 0, 1, or 2 points provided the study doesn't, partially does, or does meet the criteria respectively. Thus, the greater the score the higher the quality of evidence.

Study Type	Maximum Possible Score	Quality Threshold Scores	
		Percentage (%) of Maximum Possible Score	Quality
Quantitative	28	< 50%	Limited
		≥ 50% and < 70%	Adequate
		≥ 70% and < 80%	Good
		≥ 80%	Strong

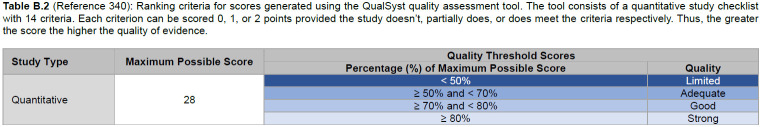

### APPENDIX C:

**Table C.1:** (Reference 280): JBI levels of evidence for effectiveness. These levels are intended to be used alongside the supporting document outlining their use and using these levels does not preclude the need for careful reading, critical appraisal and clinical reasoning when applying evidence.

Levels of Evidence – Effectiveness	
**Level 1 – Experimental Designs**	Level 1.a – Systematic review of Randomized Controlled Trials (RCTs)
	Level 1.b – Systematic review of RCTs and other study designs
	Level 1.c – RCT
	Level 1.d – Pseudo-RCTs
**Level 2 – Quasi-Experimental Designs**	Level 2.a – Systematic review of quasi-experimental studies
	Level 2.b – Systematic review of quasi-experimental and other lower study designs
	Level 2.c – Quasi-experimental prospectively controlled study
	Level 2.d – Pre-test – post-test or historic/retrospective control group study
**Level 3 – Observational – Analytic Designs**	Level 3.a – Systematic review of comparable cohort studies
	Level 3.b – Systematic review of comparable cohort and other lower study designs
	Level 3.c – Cohort study with control group
	Level 3.d – Case-controlled study
	Level 3.e – Observational study without a control group
**Level 4 – Observational – Descriptive Studies**	Level 4.a – Systematic review of descriptive studies
	Level 4.b – Cross-sectional study
	Level 4.c – Case series
	Level 4.d – Case study
**Level 5 – Expert Opinion and Bench Research**	Level 5.a – Systematic review of expert opinion
	Level 5.b – Expert consensus
	Level 5.c – Bench research/single expert opinion

**Table C.2:**

Levels of Evidence – Diagnosis	
**Level 1 – Studies of Test Accuracy Among Consecutive patients**	Level 1.a – Systematic review of studies of test accuracy among consecutive patients
	Level 1.b – Study of test accuracy among consecutive patients
**Level 2 – Studies of Test Accuracy Among Non-Consecutive Patients**	Level 2.a – Systematic review of studies of test accuracy among non-consecutive patients
	Level 2.b – Study of test accuracy among non-consecutive patients
**Level 3 – Diagnostic Case Control Studies**	Level 3.a – Systematic review of diagnostic case control studies
	Level 3.b – Diagnostic case-control study
**Level 4 – Diagnostic Yield Studies**	Level 4.a – Systematic review of diagnostic yield studies
	Level 4.b – Individual diagnostic yield study
**Level 5 – Expert Opinion and Bench Research**	Level 5.a – Systematic review of expert opinion
	Level 5.b – Expert consensus
	Level 5.c – Bench research/single expert opinion

**Table C.3:**

Levels of Evidence – Prognosis	
**Level 1 – Inception Cohort Studies**	Level 1.a – Systematic review of inception cohort studies
	Level 1.b – Inception cohort study
**Level 2 – Studies of All or None**	Level 2.a – Systematic review of all or none studies
	Level 2.b – All or none studies
**Level 3 – Cohort Studies**	Level 3.a – Systematic review of cohort studies (or control arm of RCT)
	Level 3.b – Cohort study (or control arm of RCT)
**Level 4 – Case Series/Case Controlled/Historically Controlled Studies**	Level 4.a – Systematic review of Case series/Case Controlled/Historically Controlled studies
	Level 4.b – Individual Case series/Case Controlled/Historically Controlled study
**Level 5 – Expert Opinion and Bench Research**	Level 5.a – Systematic review of expert opinion
	Level 5.b – Expert consensus
	Level 5.c – Bench research/single expert opinion

**Table C.4:**

Levels of Evidence – Economic Evaluations	
**Level 1**	Decision model with assumptions and variables informed by systematic review and tailored to fit the decision-making context.
**Level 2**	Systematic review of economic evaluations conducted in a setting similar to the decision makers.
**Level 3**	Synthesis/review of economic evaluations undertaken in a setting similar to that in which the decision is to be made and which are of high quality (comprehensive and credible measurement of costs and health outcomes, sufficient time period covered, discounting, and sensitivity testing).
**Level 4**	Economic evaluation of high quality (comprehensive and credible measurement of costs and health outcomes, sufficient time period covered, discounting and sensitivity testing) and conducted in setting similar to the decision-making context.
**Level 5**	Synthesis/review of economic evaluations of moderate and/or poor quality (insufficient coverage of costs and health effects, no discounting, no sensitivity testing, time period covered insufficient).
**Level 6**	Single economic evaluation of moderate or poor quality (see directly above level 5 description of studies).
**Level 7**	Expert opinion on incremental cost effectives of intervention and comparator.

**Table C.5:**

Levels of Evidence – Meaningfulness	
**Level 1**	Qualitative or mixed-methods systematic review
**Level 2**	Qualitative or mixed-methods synthesis
**Level 3**	Single qualitative study
**Level 4**	Systematic review of expert opinion
**Level 5**	Expert opinion
